# Annotation inconsistencies beyond sequence similarity-based function prediction – phylogeny and genome structure

**DOI:** 10.1186/s40793-015-0101-2

**Published:** 2015-11-19

**Authors:** Vasilis J. Promponas, Ioannis Iliopoulos, Christos A. Ouzounis

**Affiliations:** Bioinformatics Research Laboratory, Department of Biological Sciences, University of Cyprus, PO Box 20537, CY-1678 Nicosia, Cyprus; Division of Medical Sciences, University of Crete Medical School, GR-71110 Heraklion, Greece; Biological Computation & Process Laboratory (BCPL), Chemical Process & Energy Resources Institute (CPERI), Centre for Research & Technology Hellas (CERTH), PO Box 361, GR-57001 Thessalonica, Greece

**Keywords:** Genome-wide annotation, Genome-aware methods, Protein function prediction, Next-generation sequencing, Mis-annotation modeling, Error propagation, Genome structure, Genome evolution

## Abstract

The function annotation process in computational biology has increasingly shifted from the traditional characterization of individual biochemical roles of protein molecules to the system-wide detection of entire metabolic pathways and genomic structures. The so-called genome-aware methods broaden misannotation inconsistencies in genome sequences beyond protein function assignments, encompassing phylogenetic anomalies and artifactual genomic regions. We outline three categories of error propagation in databases by providing striking examples – at various levels of appreciation by the community from traditional to emerging, thus raising awareness for future solutions.

## Background

Genome-wide sequence annotation typically involves the prediction of gene structure and regulation, protein function and metabolic capabilities of a given species [1]. Despite (or perhaps because of) the high degree of automation, genome annotation is an error-prone process [2]. As long as annotations are revisited and curated on a continuous basis, risks associated with false positive annotations can be mitigated [3]. However, if they remain unchecked, false positives can be propagated into the public databases with detrimental effects for protein function annotation and circularly misplaced function predictions, from individual sequences to entire pathways [4].

The properties of error propagation across databases have been studied with a number of approaches and statistical models [5]. In certain cases, these mis-annotations have been documented and the source of error has been detected [6]. The strategy of sequence similarity-based function prediction is well-understood, as the general categories of intrinsic errors for genome-wide annotation have been identified [7]. Previously, we have attempted to classify those categories into a quasi-quantitative scale (named Transitive Annotation-Based Scale or TABS), in order to compare different annotation sets for a given genomic collection [1]. Partial or ambiguous annotations have been shown to generate systematic enzyme annotation errors, resulting in internal inconsistencies for EC numbers, and thus reaction and pathway assignments [4]. The situation might have been improved locally for certain data resources but is still addressed by studies tackling genome-wide annotations in systems biology efforts [8].

Herein, we examine further instances of erroneous annotations or inconsistencies beyond sequence similarity-based function prediction, involving the so-called genome-aware methods for function detection in genomic sequences [9]. These methods address the co-occurrence of proteins or protein families across a phylogeny (phylogenetic profiles) and proximal or distant gene cluster patterns across genomes (corresponding to gene clusters or gene fusions, respectively). All such methods strongly depend on high-quality gene models, assembly validation and accurate similarity detection.

We present three classes of function prediction challenges that involve an increasingly common type of sequence data, provided by next-generation short-read sequencing efforts, not previously widely recognized or appreciated in this general context^1^^,^^2^.

### Categories of errors in genome-wide sequence annotation

We discuss the three levels of errors listed above, namely the ‘classical’ similarity-based function predictions, the potentially erroneous phylogenomic anomalies and finally the most critical mis-interpretations, arising from next-generation sequencing artifacts. We have discovered these annotations in our recent research efforts, specifically the delineation of domain organization in experimentally verified gene fusion instances [9] and the functional genomics analysis of outer ring coat nucleoporins (Y-Nups) [10].

### A null example: propagation of a description line

**Fig. 1:**
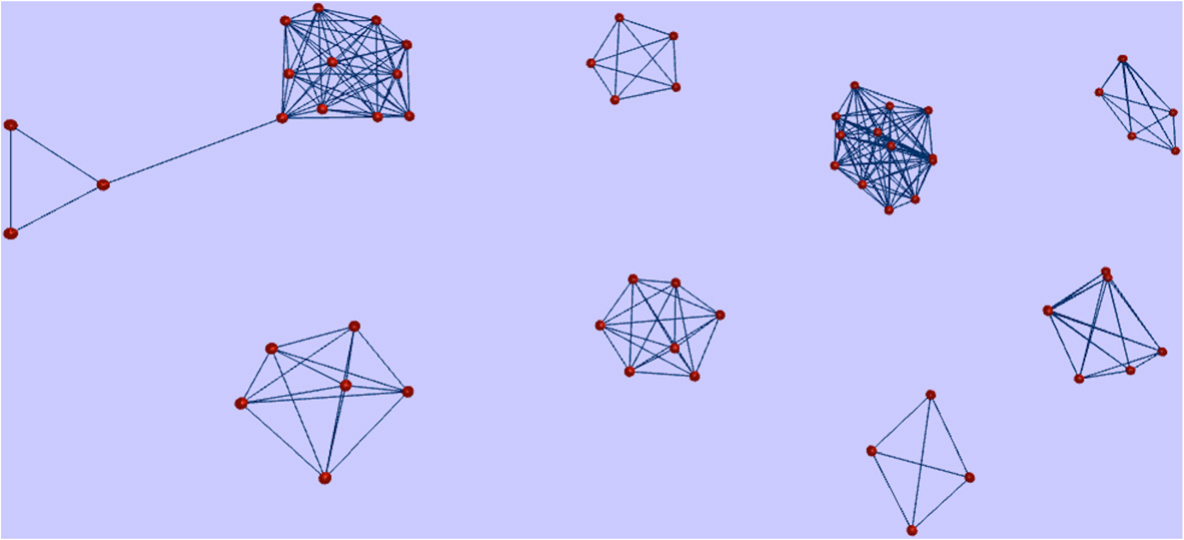
Depiction of the relationships across eight families of 62 “putaitve” proteins. Network view of sequence similarities detected by BlastP [21], generated with BioLayout [22]. Six of the eight displayed families originate from a single genome project [23]

Before discussing the traditional type of function annotation by similarity, we unveil a highly unusual case, where a typographic mistake in a description line of 99 protein database entries has emerged over time. In fact, it is rare to be able to track the source of annotation errors in an arbitrary set of sequences. Unusually, a set of proteins annotated as “Putaitve” (*sic*) can be seen as sharing structural or functional features, indicating that the simple mistyping of a description line has been copied over by automated means. Of the 99 proteins in total, 62 are clustered into 8 homologous families (clusters with more than 3 members) by sequence similarity (BLASTp e-value threshold 10^−03^) (Fig. 1).

This exceptional situation is an exemplary case of annotation transfer by sequence similarity, with a (thankfully limited) mis-annotation record that has been corrected sporadically (not shown). Unfortunately, many other, more serious cases we are aware of have been propagated through the databases for almost twenty years, making the re-naming of other protein clusters a genuine re-annotation nightmare [11]. Ways to address this general issue are community-based initiatives for model organisms, from which most other annotations are drawn using various computational methods [3], as well as manual curation of individual families within superfamilies [12]. While current sequence submission protocols of the main repositories do not readily allow modification of original entries regarding metadata including annotations – such as description lines, taxonomic classifications or sequence boundaries [13], curation efforts that encourage feedback – such as the UniProt resource [14], might benefit from external, targeted mini-annotation projects for the mitigation of this state of affairs.

### Category one: sequence-similarity function prediction

On the first level, a hand-picked set of eight protein sequence database entries are listed, which correspond to inaccurate annotations, either as under-predictions (e.g.putative) or over-predictions (e.g. delta subunit). These specific entries have been verified in the course of our recent work [10], while other misassigned entries that are not detected by sequence similarity abound (e.g. CopG family transcriptional regulator from *Sulfolobus islandicus* with accession number YP_002828985.1, and 177 other entries – not shown) (Table 1).

**Table 1:** Eight select cases of similarity-based mis-assignment

#	GI #	Accession #	Description	Species
1	19698819	gb|AAL91145.1	putative protein {Nup85}	*Arabidopsis thaliana*
2	7573329	emb|CAB87799.1	putative protein {Sec16}	*Arabidopsis thaliana*
3	296819643	ref|XP_002849880.1	protein kinase domain-containing protein {+Nic96}	*Arthroderma otae* CBS 113480
4	557867390	gb|ESS70565.1	unspecified product {Sec16}	*Trypanosoma cruzi* Dm28c
5	316978722	gb|EFV61666.1	putative ATP synthase F1, delta subunit {Nup98-96}	*Trichinella spiralis*
6	308809856	ref|XP_003082237.1	ATP-dependent RNA helicase (ISS) {Sec16}	*Ostreococcus tauri*
7	255574074	ref|XP_002527953.1	nucleotide binding protein, putative {Sec16}	*Ricinus communis*
8	443916862	gb|ELU37796.1	DUF1479 domain-containing protein {+Nup85}	*Rhizoctonia solani* AG-1 IA

Column names: #: case number, GI#: gene identifier number, Accession#: database and accession number, Description: description line, Species: species name (and strain type where available). In curly brackets within the Description field, we list the corresponding protein domains (Nup85, Nup98-96, Nic96 nucleoporins – and ancestral coatomer element 1 Sec16 (ACE1-Sec16-like); + sign: partially correct annotation, missing the domain indicated, two cases)

There are two reasons that these particular ‘genome-agnostic’ examples of similarity-based annotations need to be discussed in this broader context. First, to underline the requirement of a continuing community effort for re-annotation and refinement of critical descriptions on a genome-wide scale [1]. Second, to contrast this recurrent, traditional assignment strategy by sequence similarity to the new kinds of challenges one encounters when faced with novel types of data sources, in particular NGS short reads and their assemblies – which follows, as the main focus of this commentary.

### Category two: *a posteriori* phylogenetic anomalies

Moving on to the second level, phylogenomic patterns can be taken into account when entire protein families or classes are under consideration. The species distribution and taxonomic range of genome-level annotations can thus be taken into account. Case in point are the Y-Nups, previously verified to be phylogenetically restricted to eukaryotic genomes: in fact, extensive searches across *Bacteria* and *Archaea* have never revealed any single instance of a detectable sequence similarity beyond Eukaryotes [15].

**Fig. 2:**
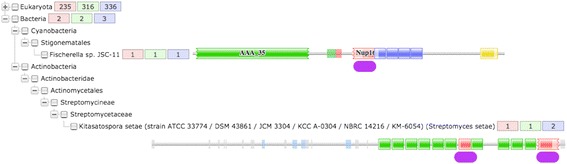
Phylogenetic distribution of nucleoporin Nup160 domains in Pfam. The collapsed eukaryotic tree with the distribution of 336 members is shown, along the bacterial branch containing two unexpected entries with 3 members (underlined by a purple oval box). These phylogenetic anomalies are present both in Pfam (PF11715) [24], as well as the corresponding UniProt entries [14]. The presence of other domains is also shown

Purported phylogenetic anomalies can be best exemplified by a particular database entry for Nup160 in Pfam (identifier: PF11715), allegedly found in *Fischerella* sp. JSC-11 (a cyanobacterial strain; UniProt accession number G6FXC6) and *Kitasatospora setae* (various actinobacterial strains; UniProt accession number E4NAP7) (Fig. 2). These entries should at least be flagged as spurious hits for nucleoporins allegedly outside the eukaryotic domain.

### Category three: rare instances of domain organization

In recent work, we have encountered a number of function annotation assignments for a handful of Y-Nups [10], exhibiting certain unexpected multi-domain architectures. These are additionally supported by Pfam domain matching, pointing towards an unusual functional role beyond the nuclear pore, and a restricted phylogenetic distribution.

As we aimed at high-accuracy assignments of these multi-domain proteins, we sought ways to eliminate potentially false positive annotations with respect to genome structure and function. We therefore devised a scheme to exclude spurious (or unsupported) hits for multi-domain organizations in our quest for functional associations of Y-Nups with other domains, using genomic and RnaSeq expression information, among other criteria [10]. The result has been that out of 27 such configurations, only 6 survive the specific exclusion criteria with very high support. These are also present in multiple species, another strong indicator for a restricted yet meaningful phylogenomic distribution.

**Fig. 3:**
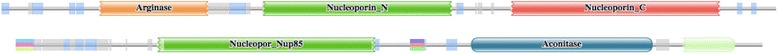
Domain organization for two unique instances of multi-domain architectures for Y-Nups. The arginase-Nup133 (Nucleoporin_C) fusion is accompanied by a Nup170-like domain in the middle (green) [top]. The aconitase-Nup75 (Nup85) fusion also contains a number of other regions of interest [bottom]. For details, please refer to the corresponding UniProt/Pfam entries, see main text for identifiers

To showcase the challenges we met during this meticulous manual annotation of approximately 3000 proteins, we selected a handpicked set of problematic database entries, which are marked by Pfam as exhibiting multi-domain architectures. We provide evidence against this claim, primarily due to next-gen/shotgun-assembly errors [10] – see also: Data Supplement 06 therein [16]. Two succinct examples are an arginase-Nup133 fusion from *Rhodotorula glutinis* ATCC 204091 (UniProt accession G0SVZ0) and an aconitase-Nup75 fusion from *Metarhizium acridum* CQMa 102 (UniProt accession E9DRH2), both of which are unique in the database and dissimilar from their closest relatives (Fig. 3).

The former case is a classical example of domain fusion without supporting evidence. We will focus on the latter case, whose annotation history can be traced. It is encoded by gene MAC_00341, which is predicted to contain two domains, the Nup75 domain at positions 244–898 and the aconitase domain at positions 900–1899: the linker sequence at positions 878–920 encodes for the C-terminal region of nucleoporin Nup75 – Figure S5 in [10]. There are no indications from any expression or short-read data that an aconitase domain follows – see also: Data Supplement 06 in [10]. Unfortunately, this mis-annotation has already propagated into other database entries since its original release in May 2010, in particular actual Nup75 homologs in other fungi, with GI numbers (date submitted): 531865436 (November 2012), 572277876 (December 2013), 597570643 (March 2014), 632915374 (April 2014), which do not appear to be homologous to aconitases, and yet they are characterized precisely as such in their description lines. While Pfam searches do not admit this description, the fact remains that the original entry is presented in domain architecture charts as a rare instance of the two domains joined into a single fusion protein. These cases should not only be treated differently deploying a number of community criteria to be agreed on, but literally blacklisted in automated function prediction (AFP) efforts. Thus, examining phylogenetic distributions of genes, proteins or protein families can also be expanded to encompass phylogenetic and genomic patterns to enhance the quality of annotation.

## Conclusions

The advent of NGS platforms has accelerated sequence discovery and function annotation to a whole new level, with the publication of shotgun assemblies of genomic sequences which are rarely completed to finished chromosomes. In this new era of shotgun sequencing and assembly, short-sequence read collections and incomplete genome surveys, additional checks are absolutely inevitable [17], to ensure that those – once considered groundbreaking – genome-aware methods achieve their full potential [9]. How would the community address the type of errors described here in a systematic manner? One solution might be by allowing for the inclusion of additional metadata to flag NGS-related projects, thus enabling the modification of annotations at the assembly and/or sequence boundary levels. Thus, annotation efforts of NGS projects will need to flag and treat differently quasi-correct genome sequences, erroneous or elliptic assemblies and inexact gene predictions. We highlight the issue for automated function prediction, which apart from the current agenda [18], should further consider any substantial NGS artifacts, as a novel challenge that has not been adequately addressed so far [19].

The sources of error might be multi-faceted and typically include both assembly and gene prediction artifacts, that truly incapacitate various automated methods. In fact, despite the expectation that more genome sequences will generally improve our predictive abilities, our experience shows that propagated errors occur deep into the raw data which render them extremely difficult to trace. Automated function prediction (AFP) thus suffers, with serious and long-standing implications for high-throughput research, genomics and systems biology. It follows that curators and programmers, collectively enriching database annotations, should relax their protein-centric views of biochemical function and start taking into account genome structure and evolution.

To go from relatively innocuous (yet very costly!) academic research activities to clinical-grade whole-genome interpretations, genome annotation inconsistencies – especially false-positives – become absolutely critical. To quote the authors of one study, “the publication of [Dr. Watson’s] genome might be regarded as a final warning of the deluge to come of incidental findings in genome-scale investigations—a downpour we have termed the incidentalome” [20]. NGS technology may produce vast amounts of data but can sacrifice quality, an essential element for genome-aware function detection methods.

We are poised to explore this approach further and, subject to adequate support, plan to automate the validation process of function prediction with additional elements at genome/transcriptome/variome/proteome levels using specific constraints deployed elsewhere [10]. We envisage less error-prone pipelines, where these artifacts are automatically corrected – or at least flagged as spurious – in domain-level annotations, providing feedback to the corresponding databases. As an emerging topic, NGS-related artifacts impacting downstream processes of annotation and function prediction might become a focal point for future AFP and SIGS meetings. The current content, if left untreated, has the potential of a ‘propagated epidemic’ across multiple entries, with unforeseeable results in skewing our understanding of genomic structure and function.

## Footnotes

1 The initial list of 31 entries in our original report [9] has been reduced to 29 – following the deletion of two entries, namely those with GI numbers 220675525 [deprecated] and 257140810 [untraceable, encoded in *Burkholderia thailandensis* E264 chromosome II, GenBank accession number CP000085.1, frame +3 coordinates: 2418321–2421305]

2 A recent report http://www.genomebiology.com/2015/16/1/99 by Triant & Pearson and the associated commentary http://www.genomebiology.com/2015/16/1/100 by Kelly & Sternberg highlight this issue as an emerging topic for protein domain definition, confounded by potential sequencing and assembly artifacts.
